# A qualitative study on oral-fluid-based HIV self-testing experiences among men in Kigali, Rwanda

**DOI:** 10.11604/pamj.2020.37.138.24353

**Published:** 2020-10-08

**Authors:** Gashema Pierre, Ariane Umutoni, Tafadzwa Dzinamarira

**Affiliations:** 1 College of Medicine and Health Sciences, University of Rwanda, Kigali, Rwanda,; 2 Department of Public Health, Mount Kenya University, Kigali, Rwanda,; 3 Department of Public Health Medicine, School of Nursing and Public Health, University of KwaZulu-Natal, Durban, South Africa

**Keywords:** HIV self-testing, men, testing experiences, Rwanda

## Abstract

**Introduction:**

there has been a global call to engage men in the fight against the HIV epidemic. Poor uptake of HIV testing services among men has been reported in most of sub-Saharan Africa where the HIV epidemic continues to be a major public health problem. HIV self-testing (HIVST) has potential to bridge the gap; however, there is a paucity of research evidence on oral-fluid-based HIVST experiences among men in Rwanda. The aim of this study was to assess oral-fluid-based HIVST experiences among men.

**Methods:**

a qualitative study based on individual interviews was employed on 21 men who voluntarily obtained oral-fluid-based HIV self-test kits and consented for follow-up post-test interviews. Interview guides explored men´s perspectives on the oral-fluid-based HIV self-screening experience. A conventional content analysis qualitative approach was adopted, entailing inductive thematic analysis.

**Results:**

the majority of participants expressed satisfaction with the experience of self-testing, citing privacy and turn-around time. Participants presented contradicting views on usability of test kits and post-test status disclosure to sexual partners. One participant reported post-test distress resulting from unexpected results.

**Conclusion:**

HIVST is attractive to men and may have potential for improving uptake of HIV testing services in this group without compromising the testing experience. Concerns on missing linkage to care and potential social harms and adverse events should not be ignored.

## Introduction

HIV continues to be a major public health concern with approximately 36.9 million people living with HIV/AIDS (PLWH) worldwide as of 2017 [1]. The Joint United Nations Programme on HIV/AIDS (UNAIDS) reported that approximately 25% of PLWH globally were unaware of their HIV status in 2017 [1,2]. HIV testing is essential in HIV epidemic control strategy as it is a gateway to prevention, treatment, care and support services [1-3]. Rwanda faces a generalized HIV epidemic and in this context the Ministry of Health (MoH) has led a systematic and sustained response, which has contributed to maintaining a stable national HIV prevalence of around 3% for the last decade [4-6]. HIV prevalence in urban settings in Rwanda is considerably higher than in rural areas, estimated at 6.2% and 2.2%, respectively [4]. The 2014 Rwanda AIDS Indicator and HIV incidence survey reported an overall incidence of 0.27 per 100 person-years (95% confidence interval (CI) 0.18-0.35) [7]. With the goal of achieving epidemic control by 2020, Rwanda is intensifying testing and treatment strategies and prioritizes evidence-based interventions.

Despite the remarkable progress of the HIV response, the scale-up of HIV testing services (HTS) has been unequal and men are being left behind [8,9]. Subsequently, men lag behind women in each of the “three 90s” diagnosis, linkage to antiretroviral therapy (ART) and viral suppression [9,10], with a more pronounced difference on the first 90. Delayed diagnosis and linkage to care and treatment results in preventable morbidity and mortality [1,11]. Data from national surveys conducted in 11 countries in sub-Saharan Africa show lower uptake of HTS among men compared with women [1]. This is also true in Rwanda, where uptake of HTS among men continues to be suboptimal; the proportion of men who have never had an HIV test in Rwanda in 2015 was much higher at 24% when compared to their female counterparts at 16% [4].

Scaling up uptake of HTS in general is a critical step toward attaining UNAIDS´ 90-90-90 targets by 2020 and ending the AIDS epidemic by 2030 but will not be effective without special attention given to men´s engagement. The World Health Organization (WHO) published the first global guidelines on HIV self- testing (HIVST) in 2016 [12], an intervention that has potential to increase uptake of HTS among hard-to-reach populations. Studies from settings where HIVST has been piloted have shown high acceptability among men [13-16]. However, there is a paucity of research evidence on post-test experiences among men in Rwanda. We sought to explore experiences of men who had voluntarily performed self-testing using Ora-Quick (Ora-Sure Technologies, Bethlehem, Pennsylvania, USA) HIVST kits in Kigali, Rwanda.

## Methods

The current study is a part of a larger study aimed at assessing acceptability of HIVST among men in Rwanda; the protocol and findings from the baseline study are under consideration for publication elsewhere. At the time of the baseline study, participants voluntarily collected Ora-Quick (Ora-Sure Technologies, Bethlehem, Pennsylvania, USA) HIVST kits and consented for post-test follow-up interviews. A trained study team of health care providers set up a private room at the study sites where men were invited to learn about HIVST and participate in the study. At baseline, we conducted interviews to assess perspectives of men toward HIVST. Consenting men were offered HIVST to take home and a manufacturers´ package insert with pictorial instructions on how to perform a self-test were provided in English. All our study participants consented for a follow-up interview to assess their perspectives on the testing experience with HIVST kits.

**Setting and study population:** interviews were conducted in a private room at each of the study sites. We purposively selected one public youth center (Kimisagara Youth Center), one private (Mount Kenya University, Rwanda) and one public university (University of Rwanda) as study sites based on convenience for the researchers. Our study population was men attending the selected sites.

**Sampling and eligibility:** at baseline, we used convenience sampling to recruit participants. Men who self-reported to be unaware of their HIV status at the time of the study were invited to participate. Recruited participants provided consent for a follow-up interview at the same study site three months later. Follow-up interviews were scheduled three months from day when participants obtained HIVST kits. The researchers chose to perform follow-up interviews three months post-first-test to allow sufficient time to assess repeat HIV testing and linkage to care and treatment for HIV positive cases. Our participants were routinely attending the study sites; as such they did not incur any additional cost for study participation.

**Participants:** our participants were 21 men aged 18 - 39 initially recruited during the period 1^st^ to 28^th^ February 2019 and followed up during the period 6^th^ to 17^th^ May 2019. Of the participants, 12 were recruited at Kimisagara Youth Center, while 6 and 3 were recruited from University of Rwanda and Mount Kenya University, respectively. Only 4 were married, while 9 were educated up to tertiary level. All participants had reported being unaware of their HIV status and reported having had at least one sexual partner at the time of the baseline study. The methodology and results of the baseline study are under consideration for publication elsewhere. At baseline, participants received routine health education for HIV/AIDS and HIVST-specific education using Ora-Quick (Ora-Sure Technologies, Bethlehem, Pennsylvania, USA) package insert. In accordance with the Rwanda guidelines for HIVST, recommendations to contact the HIVST toll free number 114 for a negative HIVST outcome and visit a health facility for verification testing and additional care and treatment services were provided at baseline study.

**Figure 1:**
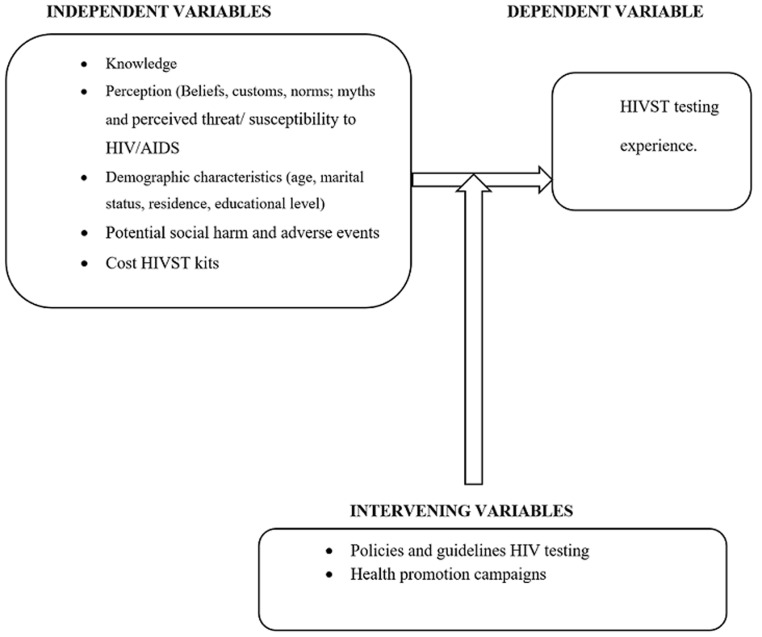
conceptual framework for the study

**Data collection and analysis:** phenomenology, an approach that is suited for qualitative research, guided how in-depth interviews were conducted using a semi-structured guide that contained open-ended questions. Phenomenology focuses on the commonality of a lived experience within a particular group. In this study context, the lived experience was the HIV self-screening process and the particular group was of men. This approach has been proven to allow qualitative researchers to construct the universal meaning of the event, situation or experience and arrive at a more profound understanding of the phenomenon. The interview guide variables were informed by themes from out baseline study. The methodology and interpretation of data were guided by a conceptual framework (Figure 1) developed by the researchers based on the research question. We piloted the interview guide with two participants who had previously used HIVST kits and used insights generated to revise the study tool before actual data collection. Of the 22 initially recruited participants, only one was a loss to follow up as the study team failed to make contact with the participant. Ariane Umutoni, a trained HIV counsellor who received additional study-specific training, conducted interviews. The interviews were scheduled at the convenience of the participant. Participants were contacted via telephone to schedule an appropriate time for the interview. Interviews were conducted in Kinyarwanda and lasted between 15 and 40 minutes. We conducted interviews on 21 men who had been recruited in the baseline study.

All tape-recorded interviews were transcribed word-for-word by a professional transcriber. The transcribed text from each informant was translated from Kinyarwanda to English. First and second authors read all the transcripts while playing the audio tapes to ensure the accuracy of the transcription. Along with observation notes taken during each interview, first (Gashema Pierre) and second (Ariane Umutoni) authors coded the transcripts for inductive thematic analysis. Gashema Pierre and Ariane Umutoni developed the preliminary coding scheme and applied it to all transcripts. Tafadzwa Dzinamarira independently reviewed several transcripts and noted independent observations. A final coding framework was developed through discussion. An inductive approach to thematic analysis was employed. This involved allowing the data to determine the themes.

**Measures to ensure trustworthiness:** we used prolonged engagement [17,18] to ensure credibility and pilot testing of the interview guide [19,20] to ensure dependability. Data analysis was based on the naturalistic paradigm with a conventional content analysis where coding categories are derived directly from the text data. We chose this approach as it limits researcher bias due to pre-conceived ideas or theoretical perspectives [21].

**Patient and public involvement:** this study recruited members of the general public at three institutions in Kigali, Rwanda. All participants provided consent prior to any study procedures.

**Ethical considerations:** this study was approved by the University of Rwanda, College of Medicine and Health Sciences Institutional Review Board (approval number: 094/CMHS IRB/2018). Permission was obtained from the principals at tertiary institutions and the coordinator at Kimisagara Youth Center prior to the conducting of this study. All study participants also signed an informed consent prior to participating in the study. All the quotations are anonymized as participant 1 - 21 in the results section, to protect participants´ identities. Pseudonyms were used in the encrypted transcripts to maintain confidentiality.

## Results

Four themes emerged from data analysis. Here we present our findings on men´s oral-fluid-based HIVST experiences as described by the participants.

**Clarity of test kit instructions and procedure:** the majority of the study participants expressed satisfaction with the experience of self-testing, citing privacy, robustness and turn-around time. However, our participants reported mixed feedback on clarity of the test kit instructions and procedure. Some reported that the package insert instructions were clear and found the questions and answers section of the insert to be very helpful. On the other hand, others felt it may be worth considering a translated package insert in French and/or Kinyarwanda, the local language. From our findings, it emerged that the major challenge to understanding the test kit instructions and procedure was mainly language barriers as some participants could not understand some of the English terms. “All things (overall test procedure and package insert) were understandable, I had no challenges, however, fitting the tube in the stand was somehow not easy. Maybe that can be improved” (participant 2). “(...) you know we are changing from French to English in Rwanda. Some of the big English words were confusing. It could be better if the package insert also came with French and even Kinyarwanda version” (participant 4).

**Results disclosure, linkage to care and treatment post-HIVST:** some of our participants reported to have not disclosed their HIV self-test results to their sexual partners. Of these participants, most were coming from a school of thought where it was not necessary to divulge a negative result. For those that disclosed, participants cited the need for partner support and health education provided during baseline study as facilitators to disclosure. For post-test counselling, linkage to care and treatment, we asked participants if they contacted a health facility or a health care provider via telephone or visited a health facility. In their responses, most of our participants felt there was no need to contact or visit a health facility in the case of a negative self-test result. None of the participants self-reported a positive HIV self-screening result. “I think one needs counselling in the case of a positive (result). Once you see that [HIV] negative result, you are relaxed. I did not think of that (...) maybe I will just do another self-test now that it is three months later and be sure” (participant 3). “I did not find it necessary to do that [contact health care provider or visit health care facility]. I tested negative so what was the point? I thought this [HIV] self-test kit was supposed to help us avoid going there [health facilities]” (participant 9).

**Social harms and adverse events:** one participant reported distress with the testing experience. The participant reported observing a control line and a broken (half) test line. This prompted extreme fear and worry as the participant was sure to have not been at risk of HIV. “This test disappointed me. I performed the test and it marked positive with a broken test line while was sure of my HIV status. I went to the clinic and my test was negative. I can´t use this test and I can´t recommend [it]. I imagine if I was that weak person, I could have committed suicide” (participant 15).

**Men´s recommendations and preferences on distribution of oral-fluid-based HIVST kits:** in general, men recommended the need for more health education on HIVST. Concerns were raised on lack of awareness. Most participants recommended that HIVST kits should be sold over the counter or availed in the same manner as are condoms where men can access them discretely. The rest reported a mix of home delivery/visit, call centers for ordering test kits and collection from local leaders as preferences for distribution of self-test kits. Most men cited fear of stigma and long waiting times as an important reason for avoiding collection of self-test kit at health facilities. “They should emphasize this test like what has been done on condoms. Most of men I spoke to don´t know about it” (participant 20). “I think they should make people aware of it, like advertisement, mobilization and it should be everywhere. This program would help so many men; Ministry of Health needs to put more effort making people know about it [HIVST]” (participant 21). “Ministry of Health needs to distribute these HIV self-test kits in supermarkets, pharmacies at low price, or giving for free like they do for condoms (...) some guidance on how to perform the test will be needed” (participant 6).

## Discussion

In Uganda, a similar study reported general positive HIVST experiences among men [22]. The majority of participants found the package insert instructions to be clear and easy to follow. However, consistent with the findings of Peck *et al*. [23], some of our participants reported difficulties in understanding test instructions and reading results. Some of our participants felt this can be improved by translation of instructions to the local language. This is consistent with earlier work by Tonen-Wolyec *et al*. [24], van Rooyen *et al*. [25] and Grésenguet *et al*. [26]. Closer to home in Kenya [27], other scholars have recommended health education campaigns and improved instructions particularly for men. This study provided mixed perceptions on where participants would recommend to buy or collect HIV self-test kits. Participants were comfortable with over-the-counter purchase of HIVST kits, similar to findings from other studies [28], while others recommended home delivery, similar to findings from Indravudh *et al*. [29]. An emerging sub-theme was that participants were comfortable with collection or purchase of HIVST kits anywhere other than health facilities that are routinely offering HTS. Some of the reasons mentioned were fear of stigma and long waiting times. Similar findings were revealed in a recently published systematic review on men´s perspectives on HIVST [30].

The ultimate goal of HIVST is to ensure people are linked to care and treatment. Half of our participants reported to at least contacting a health facility via telephone post-HIVST. Others believed that visits to health care facilities were only for those who tested HIV positive. However, a few of our participants were reluctant to visit a health facility regardless of HIVST result. This is consistent with findings from a study done in Malawi [31]. HIVST and missing the linkage has been well researched [32]. The participants recommended a call center and home visits by a health care provider. Similar to collection or purchase of self-test kits, aversion to health facilities seemed to be a key driving factor. This is consistent with findings from male and youth groups in South Africa [33] who recommended home visits for follow-up counselling.

A global qualitative systematic review [34] revealed that positive HIVST results did not lead to adverse outcomes (suicide, violence) in the 18 studies reviewed but had emotional impact in other contexts. Our participants reported no adverse events; however, one case of emotional impact was reported. Emotional concerns have been raised elsewhere due to fear of incorrect results and invalid test results [35]. Experience with HIVST kits encouraged some of our participants to disclose their status to their partners. In addition, HIVST has been reported to facilitate HIV status disclosure to sexual partner in other studies [22,36,37]. There is a growing body of evidence on feasibility of HIVST uptake among men [27-29,31] and positive testing experience feedback [35-37]. This provides reassurance on the possibility of HIVST intervention´s ability to bridge the gap in the poor uptake of HTS among men. Concerns still need to be addressed [32]. Men have been identified as a priority population for HIV testing services [38]. Stakeholders in Rwanda may draw information crucial in planning of HIVST intervention expansion based on post-test experience responses provided by our participants.

The current study has limitations. Firstly, HIV self-testing is a test one conducts in private. The researchers have no way of verifying that the participants actually used the test kit. Secondly, the quality of data and findings of a qualitative study are highly subjective. However, we used a rigorous thematic content analysis process where data were generated directly from participant responses, which minimized the risk of preconceived ideas from the researchers. Third, all our participants fell in the age group of 18-39, not representative of adult men in Kigali, Rwanda. This limits generalizability of results. We recommend further substantive quantitative research to inform policy.

## Conclusion

In general, positive HIV self-testing experiences feedback was provided by our participants. HIVST may have potential to increase the uptake of HTS without compromising the testing experience. Concerns on missing linkage to care and potential social harms and adverse events should not be ignored.

**Data availability:** the interview transcripts used to report findings in this study are available upon reasonable request in writing from the first author. Further, permission will be sought from the University of Rwanda, College of Medicine and Health Sciences Institutional Review Board.

### What is known about this topic

- There is a plethora of research evidence on low uptake of HIV testing services among men;
- While multiple interventions have been implored, HIV self-testing presents as a promising intervention capable of bridging the gap.

### What this study adds

- To our knowledge, this is the first study that explores and reports on HIV self-testing experiences and among men in Rwanda. This information is critical to inform interventions that aim to improve uptake of HIV self-testing among men in Rwanda;
- The study reveals that men in Rwanda perceive oral fluid-based HIV self-test kits as acceptable while also highlighting some important concerns on missing linkage to care and potential social harms associated with this testing approach.
